# *VvVHP1; 2* Is Transcriptionally Activated by VvMYBA1 and Promotes Anthocyanin Accumulation of Grape Berry Skins via Glucose Signal

**DOI:** 10.3389/fpls.2017.01811

**Published:** 2017-10-20

**Authors:** Tianyu Sun, Lili Xu, Hong Sun, Qianyu Yue, Heng Zhai, Yuxin Yao

**Affiliations:** State Key Laboratory of Crop Biology, Key Laboratory of Biology and Genetic Improvement of Horticultural Crops (Huang-Huai Region), Ministry of Agriculture, College of Horticulture Science and Engineering, Shandong Agricultural University, Tai’an, China

**Keywords:** *VvVHP1; 2*, VvMYA1, transcriptional regulation, anthocyanins, grape berry, glucose signal

## Abstract

In this work, four *vacuolar H^+^-PPase* (*VHP*) genes were identified in the grape genome. Among them, *VvVHP1; 2* was strongly expressed in berry skin and its expression exhibited high correlations to anthocyanin content of berry skin during berry ripening and under ABA and UVB treatments. *VvVHP1; 2* was transcriptionally activated directly by VvMYBA1, and *VvVHP1; 2* overexpression promoted anthocyanin accumulation in berry skins and *Arabidopsis* leaves; therefore, *VvVHP1; 2* mediated VvMYBA1-regulated berry pigmentation. On the other hand, RNA-Seq analysis of WT and transgenic berry skins revealed that carbohydrate metabolism, flavonoid metabolism and regulation and solute carrier family expression were the most clearly altered biological processes. Further experiments elucidated that *VvVHP1; 2* overexpression up-regulated the expression of the genes related to anthocyanin biosynthesis and transport via hexokinase-mediated glucose signal and thereby promoted anthocyanin accumulation in berry skins and *Arabidopsis* leaves. Additionally, modifications of sugar status caused by enhanced hexokinase activities likely play a key role in *VvVHP1; 2-*induced sugar signaling.

## Introduction

There are two phylogenetically distinct H^+^-PPases in plant cells. Type I H^+^-PPases are K^+^ sensitive, and type II H^+^-PPases are K^+^ insensitive but extremely Ca^2+^ sensitive. Plant type I H^+^-PPases are usually located on the vacuolar membrane and are considered to be *bona fide* vacuolar markers (Rea et al., 1992; Maeshima, 2000); thus, they are also named vacuolar H^+^-PPases (VHPs). Additionally, increasing evidence indicates that VHPs are localized on the plasma membrane of phloem cells in *Arabidopsis* and other plant species (Khadilkar et al., 2016; Regmi et al., 2016); however, greater localization of AVP1 at the vacuolar membrane compared with the plasma membrane has been reported (Segami et al., 2014). VHP1 enzyme consists of a single 80 kDa polypeptide (Maeshima, 2000). Type II H^+^-PPases localize and function in the Golgi apparatus, and their total amount in tissues is very low, less than 0.2% of the type I H^+^-PPase (Segami et al., 2010). Therefore, type I VHPs and H^+^-ATPase have been predicted to be the key enzyme for vacuolar acidification.

Multiple functions of VHP1 have been reported in plants. VHP1 localizes in the tonoplast and acidifies vacuoles in plant cells through the coupling of pyrophosphate (PPi) hydrolysis and active proton transport, and its function in generating H^+^ motive force is comparable to vacuolar H^+^-ATPase (Zhen et al., 1997). VHP1 is a critical factor in the regulation of cell turgor through its effects on secondary active transport of inorganic ions, organic acids, sugars and other osmoregulation substances across the tonoplast (Gaxiola et al., 2007). It has also been reported that AVP1 localized at the plasma membrane of the sieve element-companion cell complexes functions as a synthase and that this activity is critical for the maintenance of pyrophosphate homeostasis required for phloem function (Pizzio et al., 2015); phloem-specific *AVP1* knockdown and overexpression demonstrated that *AVP1* increases sucrose phloem loading, transport, and unloading into sink organs in *Arabidopsis* (Pizzio et al., 2015; Khadilkar et al., 2016). Additionally, VHP1 is also reported to affect auxin-dependent organogenesis and morphogenesis (Li et al., 2005; Gaxiola et al., 2007), to facilitate the gluconeogenesis during early seedling development (Ferjani et al., 2011), and to improve nutrient use efficiency (Li et al., 2014). Compared with metabolism modifications caused by VHP1s, the most reported functions of VHP1s are to enhance abiotic stress tolerance and to promote plant growth. It has been demonstrated that overexpression of *AVP1* and other plant *VHP1* genes can increase both salt and drought tolerance in diverse systems, including tobacco (Li et al., 2014), *Arabidopsis* (Gaxiola et al., 2001), alfalfa (Bao et al., 2016), and apple (Yao et al., 2011). Overexpression of *VHP1* improves growth in various plant species (Li et al., 2005, 2014; Lv et al., 2008), and loss-of-function mutants (fugu5s) of *AVP1* in *Arabidopsis thaliana* have post-germinative developmental defects (Asaoka et al., 2016); VHP1 has been considered as a yield-enhancing factor (Khadilkar et al., 2016).

In grape cells, anthocyanins are synthesized at the cytosolic surface of the endoplasmic reticulum by multiple enzymes via the flavonoid pathway (Boss et al., 1996). Most of the genes encoding enzymes in the anthocyanin biosynthesis pathway have been well studied in grapevines (Kühn et al., 2013). Of those enzymes, UDP glucose:flavonoid 3-*o*-glucosyltransferase (UFGT), which glycosylates anthocyanidins, is critical for anthocyanin biosynthesis in grape berry skins (Boss et al., 1996; Ford et al., 1998). The expression of this gene is transcriptionally regulated by the VvMYBA1 and VvMYBA2 transcription factors, either of which can regulate the color of the grape berry, in *Vitis vinifera* grapes (Kobayashi et al., 2004; Walker et al., 2007). *VvMYBA1* and *VvMYBA2* are closely clustered in a single locus that is referred to as the berry color locus; rare mutations in the two adjacent *VvMYBA* genes are essential for the genesis of white grapes (Walker et al., 2007). In parallel with their biosynthesis in the cytosol, anthocyanins are rapidly transported into the vacuole for storage. To date, two major transport models have been proposed: membrane vesicle- and membrane transporter-mediated transport (Zhao and Dixon, 2010). Concerning the transporter-mediated model, ATP-binding cassette (ABC) transporters and multidrug and toxic extrusion (MATE) transporters participate in anthocyanin transport across the tonoplast (Yazaki, 2005; Francisco et al., 2013). ABCC1, an ABC transporter, mediates the transport of glucosylated anthocyanidins, which is dependent on GSH (tripeptide glutathione) without the formation of an anthocyanin-GSH conjugate in grapevine (Francisco et al., 2013). Two grapevine MATEs, AM1 and AM3, mediate the specific transport of acylated anthocyanins in the presence of MgATP (Gomez et al., 2009).

To date, the role of *VHP* genes in regulating fruit pigmentation and the mechanism involved remains unknown. In this study, the grape *VvVHP* isoform genes were identified at the whole genome level, and their associations with berry pigmentation were evaluated. Additionally, the function of *VvVHP1; 2* in promoting pigmentation was identified by its overexpression in berry skins and *Arabidopsis* leaves. Moreover, the mechanism underlying the regulation of *VvVHP1; 2* on pigmentation was explored by investigating the interaction of VvMYBA1 and *VvVHP1; 2* as well as *VvVHP1; 2-*induced glucose signal.

## Materials and Methods

### Plant Materials, Culture Conditions and Treatments

Grape berries were collected at different days after veraison (DAV) from 5-year-old ‘Kyoho’ grapevines (*V. vinifera* × *labrusca*). All berries were collected from the middle position of the clusters. Freshly separated skins were frozen in liquid nitrogen immediately and stored at -70°C for further determinations. Grape berries collected at -15 DAV were used for the ABA and UVB treatments. For ABA treatment, the berries were soaked in distilled water (control) or 100-μM ABA with a 14/10-h (light/dark) photoperiod at approximately 260 μmol m^-2^ s^-1^ at 25°C for 10 days. For UVB treatments, the spike-stalks of detached grape clusters were soaked in water and berries were continuously exposed to UVB (320 nm) generated by a UVB-emitting diode panel for 156 h. The berry skins were separated at different treatment time points and treated as described above. Berry skins were separated from the sterilized berries at veraison were *in vitro* cultured on MS medium supplemented with 10 mM mannoheptulose, a specific inhibitor of hexokinase (HXK), or mannitol as an osmotic control to evaluate the effects of the inhibition of HXK on gene expression.

Tobacco (*Nicotiana benthamiana*) plants were used for transactivation assays. Tobacco plants were grown in a tissue culture chamber at 25°C under a 16-h light (approximately 260 μmol m^-2^ s^-1^) regime daily for 60 days until transient transformation. WT and transgenic *Arabidopsis* seedlings grown on organic growth media were watered with 10 mM mannoheptulose or mannitol at 3-day intervals, and 3-week-old leaves were sampled to determine anthocyanin and gene expression.

### Identifications and Sequence Analysis of *VvVHP* Gene Family in Grape

Taking the *Arabidopsis* VHP proteins encoded by *AVP1* (*At1g15690*), *AtVHP2;1* (*At1g78920*) and *AtVHP2;2* (*At1g16780*) as references, protein blast analysis against grape genomic data^1^ were performed. Sequence alignments of different *VHPs* were performed using DNAman software (V8.0). An unrooted phylogenetic tree was generated with the neighbor-joining method using DNAman software (V8.0). Motif analysis was performed online^2^.

### Genetic Transformation of *VvVHP1; 2* and Its Promoter into *Arabidopsis*

The ORF of *VvVHP1; 2* and its promoter *P_V HP1;2_* were isolated using the primer pairs VHP-O1 and VHP-P1, respectively (**Supplementary Table S1**). For *Arabidopsis* transformation, the vectors of *35S:VvVHP1; 2* and *P_V HP1;2_-*GUS were constructed and the corresponding plasmids were transferred into Columbia-0 by a floral dip method mediated with *Agrobacterium* strain GV3101 (Clough and Bent, 1998).

### Binding Assay Using a Yeast One-Hybrid System

A protein binding experiment was performed using the Matchmaker^TM^ Gold Yeast One-Hybrid Library Screening System (Clontech, Mountain View, CA, United States). Three tandem copies of MBSII elements were synthesized and inserted into pAbAi vector. The resultant plasmid was introduced into the yeast strain Y1HGold, generating an MBSII-specific bait-reporter yeast strain. The background AbA^r^ expression of the bait-reporter strain was tested. The ORF of *VvMYBA1* was amplified using the primer pair of MYB-ORF1 (**Supplementary Table S1**) and fused in-frame with the GAL4 activation domain of the one-hybrid vector pGADT7, generating pGAD-MYBA1. The MBSII-specific bait-reporter strain was transformed with pGADT7 or pGAD-MYBA1 plasmid, and a mutant MBSII (mMBSII) was used as a negative control. The detailed yeast one-hybrid procedure was performed according to the user manual of this system (Clontech, Mountain View, CA, United States).

### Transactivation Assay

The synthesized tandem sequences containing MBSII and mMBSII were fused to the upstream of the *35S* minimal promoter in pRI101-GUS (Takara, Dalian, China) to generate the MBSII and mMBSII mini-GUS plasmids. The promoter sequence of *VvVHP1; 2*, -2100 bp upstream of ATG, was cloned by the primer pair of VHP-P2 (**Supplementary Table S1**) and used to replace *35S* promoter of pRI101-GUS, and the ORF, amplified by the primer pair VHP-O1 (**Supplementary Table S1**), was inserted upstream of *GUS*, generating the *P*_V HP1;2_::*VHP1;2-GUS*plasmid. The ORF of *VvMYBA1* was inserted into pRI101, replacing *GUS*, which generated the *35S:MYBA1* plasmid. The constructed plasmids were introduced into *Agrobacterium tumefaciens* strain GV3101. The *Agrobacterium*-mediated transient transformation of tobacco leaves was performed as previously described (Yang et al., 2000). The *agro*-infiltrated plants were maintained in a moist chamber at 25°C for 48 h. GUS histochemical staining and fluorometric analysis were performed according to the methods of Jefferson et al. (1987). The GUS activity was calculated as nmol of 4-Methylumbelliferone (4-MU) per mg protein per minute under controlled conditions.

### Construction of the Viral Vector and Their Administration to Grape Berries

The viral vector pIR was used and the vector construction and transfection were performed as described by Peretz et al. (2007). The pIR-GUS vector was used as the control vector and to evaluate whether IL-60 system worked in grape berries. To construct the *VvVHP1; 2* overexpressing vector, the full ORF of *VvVHP1; 2* were amplified from grape fruit cDNA using the primer pair VHP-O3 (**Supplementary Table S1**). The resultant PCR products were TA cloned into the plasmid pMD-18T. GUS gene in the pIR-GUS vector was replaced by the ORF of *VvVHP1; 2* by digesting with *Bam*H I and *Xba* I and ligating, and the resultant construct was designated pIR-VHP1;2. The pIR-GUS and pIR-VHP1;2 vectors were transformed into *Escherichia coli* cells. The plasmid DNA was extracted from the propagated *E. coli* cells under ampicillin selection. The spike-stalks of the grape clusters at -30 DAV were punctured with a hypodermic needle. A capillary tube was inserted into the resultant hole, and approximately 200 ng of DNA (in 50 μl) was pipetted into the tube until fully soaked up by the grape clusters. The injection was performed three times at 3-day intervals. The IL-60-BS vector was used as a helper plasmid.

### RNA-Seq and Quantitative Real-time PCR (qRT-PCR)

Total RNAs were extracted using TRIzol reagent (Invitrogen, Carlsbad, CA, United States) and mRNAs were purified from total RNAs using poly-T oligo-attached magnetic beads. Sequencing libraries were generated using NEBNext^®^ Ultra^TM^ RNA Library Prep Kit for Illumina^®^ (#7530L, NEB, United States) following the manufacturer’s recommendations, and index codes were added to attribute sequences to each sample. RNA concentration of library was measured using the Qubit^®^ 3.0 Flurometer (Life Technologies, Carlsbad, CA, United States), and insert size was assessed using the Agilent Bioanalyzer 2100 system (Agilent Technologies, Santa Clara, CA, United States). After RNA concentration of library and insert size were assessed, the clustering of the index-coded samples was performed on a cBot cluster generation system using HiSeq PE Cluster Kit v4-cBot-HS (Illumina) according to the manufacturer’s instructions. After cluster generation, the libraries were sequenced on an Illumina Hiseq 4000 platform and 150 bp paired-end reads were generated. Clean reads were assembled into transcripts using Cufflinks with the grape genome^3^ as a reference. Unigene expression levels were quantified using reads per fragments per kilobase of transcript per million mapped reads (RPKM). Unigenes differentially expressed between two samples were screened using false discovery rate <0.05 and absolute log_2_ (fold changes) ≥1 as the threshold. The three replicates for RNA-Seq were conducted for each sample. Real-time quantitative PCR was performed using SYBR Green MasterMix (SYBR Premix EX Taq TM, Dalian, China) on a BIO-RAD iQ5 (Hercules, CA, United States) instrument, and the primers were listed in **Supplementary Table S1**.

### Enzyme Extraction and Activity Assays

Proteins of tonoplast membranes were isolated and VHP activities were determined according to our previously reported method (Yao et al., 2011). Total protein was extracted from 1 g fresh berry skins and 0.5 g *Arabidopsis* leaves and HXK activity was measured by an enzyme-linked assay according to the method of Schaffer and Petreikov (1997). Determinations were made in a 1 ml reaction mixture containing 30 mM Hepes-NaOH (pH 7.5), 1 mM MgCl_2_, 0.6 mM EDTA, 9 mM KCl, 1 mM NAD, 1 mM ATP, 1 unit of NAD-dependent glucose-6-phosphate dehydrogenase and 50 μl extracts. The reaction was initiated with 2 mM glucose. Reactions were carried out at 37°C and A340was monitored continuously, and HXK activity was calculated from the slope of the resulting curve. HXK activity was normalized to the protein content of the extracts, and the unit was expressed as the amount of enzyme required to phosphorylate 1 nmol of glucose at 37°C for 1 min.

### Metabolite Assays

Glucose and fructose were extracted with 95% methanol and determined using a capillary electrophoresis system (Beckman P/ACE, Fullerton, CA, United States). The detailed extraction and determination methods were described in our previous study (Li et al., 2013). Glucose 6-phosphate (Glu 6-P) and fructose 6-phosphate (Fru 6-P) were extracted and assayed spectrophotometrically from 1 g fresh berry skins and *Arabidopsis* leaves according to the method previously described by Tobias et al. (1992). Relative anthocyanin levels were determined according to our previous method (Li et al., 2013). The relative value was calculated by the formula OD = A530–0.25^∗^A657, and the unit was expressed as U/g FW.

## Results

### Identification and Sequence Analysis of *VvVHP* Genes

**FIGURE 1:**
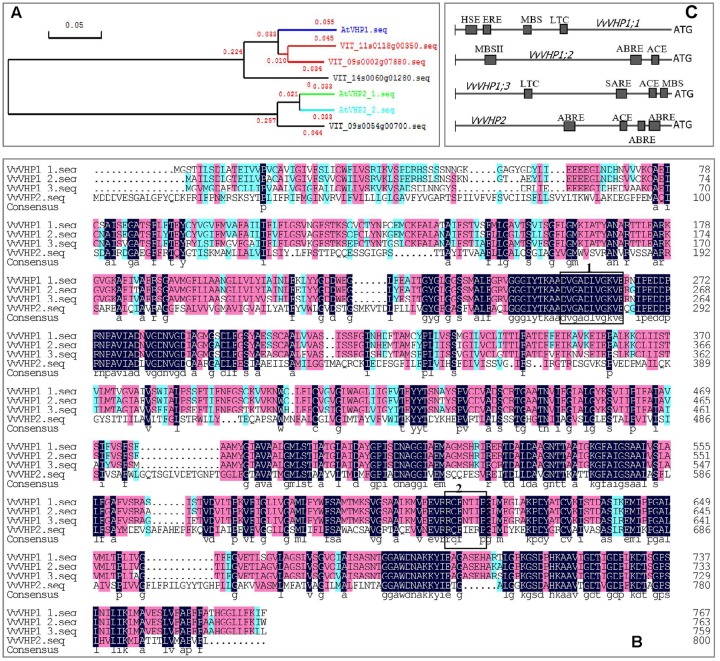
Phylogenetic tree of grape and *Arabidopsis* VHPs **(A)**, amino acid alignment of four VvVHPs **(B)**, and *cis*-acting elements in the promoters of *VvVHPs* **(C)**. In **(B)**, regions marked in boxes indicate putative pyrophosphate-binding motif DVGADLVGKVE (box 1) and putative 14-3-3 protein-binding sequence RQFNTIP (box 2). MBSII, MYB binding site involved in flavonoid biosynthetic genes regulation; MBS, MYB binding site involved in drought-inducibility; ABRE, abscisic acid responsiveness element; ACE, light responsiveness; HSE, heat stress responsiveness; MBS, MYB binding site involved in drought-inducibility; ERE, ethylene responsive element; SARE, salicylic acid responsive element.

Using *Arabidopsis thaliana* AtVHPs as reference sequences, 4 counterparts were found in the grape genome, i.e., VIT_09s0002g07880, VIT_11s0118g00350, VIT_14s0060g01280 and VIT_09s0054g00700, designated VvVHP1;1, VvVHP1; 2, VvVHP1;3 and VvVHP2, respectively, in light of their sequence similarity to their *Arabidopsis* counterparts (**Figure 1A**). VvVHP1; 2 and VvVHP1;3 were located in chr 11 and 14, respectively, and VvVHP1;1 and VvVHP2 were on chr 9. VvVHP1;1 shared the highest sequence similarity of 89.2% to AVP1. The three VvVHP1 proteins shared 90.1% sequence similarity and exhibited less than 71.7% similarity to VvVHP2 (**Figure 1A**). Additionally, a highly conserved putative pyrophosphate-binding motif, DVGADLVGKVE, was found in the four VvVHP proteins; in contrast, a putative 14-3-3 protein-binding sequence was found only in the three VvVHP1s (**Figure 1B**). On the other hand, promoter sequence analysis identified different cis-acting elements for the four genes, suggesting their different roles in response to stresses, hormones and light (**Figure 1C**).

### Expression Analysis of *VvVHPs* in Different Tissues

**FIGURE 2:**
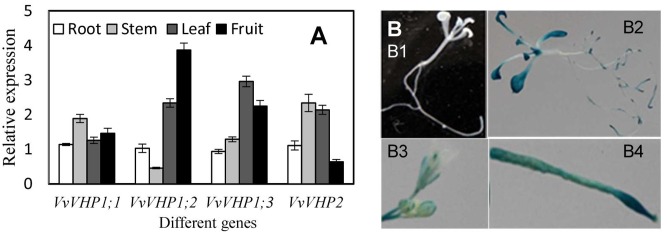
Expression levels of *VvVHPs* in different grape tissues **(A)** and expression of *GUS* driven by *VvVHP1; 2* promoter in transgenic *Arabidopsis* seedlings **(B)**. In **(B,B1)**, wild type *Arabidopsis* seedling; **(B2)** transgenic *Arabidopsis* seedling; **(B3)** inflorescence; **(B4)** silique.

The four *VvVHPs* were expressed in all examined tissues including root, stem, leaf and fruit. However, their expression levels varied with tissues. *VvVHP1; 2* was expressed strongly in fruit while weakly in stem. *VvVHP1;3* was primarily expressed in leaf. *VvVHP2* showed high expression levels in stem and leaf but low levels in fruit. In contrast, *VvVHP1;1* exhibited similar expression levels in different tissues (**Figure 2A**). On the other hand, a 2100-bp promoter sequence of *VvVHP1; 2* was obtained, and its function was verified by the expression of a *GUS* gene driven by this sequence in *Arabidopsis*. Additionally, strong GUS expression was seen in *Arabidopsis* root, leaf, inflorescence and silique; in contrast, GUS expression was weak in stem (**Figure 2B**).

### Anthocyanin Accumulations and Expression Analysis of *VvVHPs* during Berry Ripening and Under UVB and ABA Treatments

**FIGURE 3:**
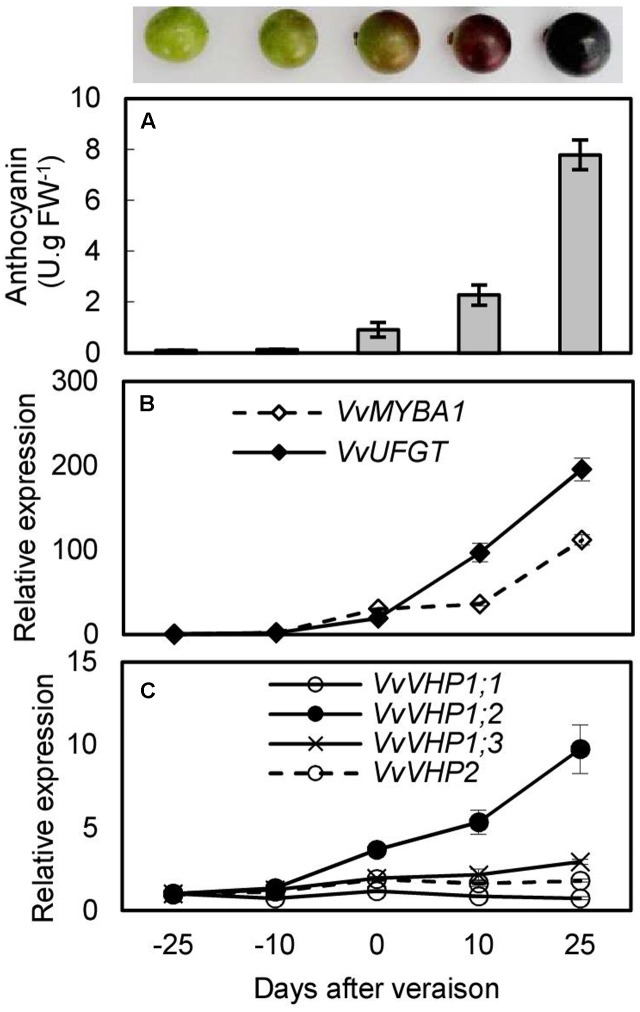
Anthocyanin content **(A)** and expression of *VvMYBA1*, *VvUFGT* **(B)** and *VvVHPs* **(C)** during berry ripening of ‘Kyoho’ grape. Values represent the means ± SD of 3 replicates.

**FIGURE 4:**
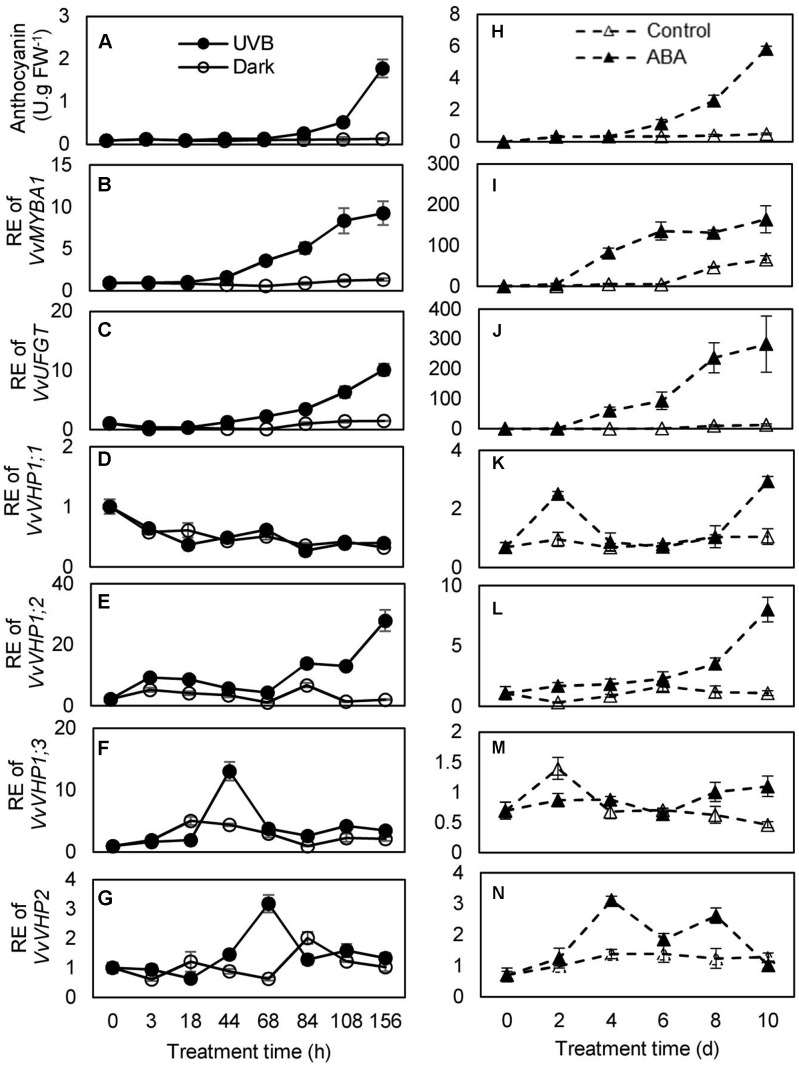
Anthocyanin accumulation in the berry skins of the ‘Kyoho’ grape **(A,H)** and the expression of *VvMYBA1* **(B,I)**, *VvUFGT* **(C,J)**, and *VvVHPs* **(D–G,K–N)** following the treatments with UVB and 100 μM ABA. RE, relative expression. Values represent the means ± SD of 3 replicates.

To investigate whether there was an association between anthocyanin accumulation and expression of *VvVHPs*, anthocyanin content and gene expression levels were detected during berry ripening and under UVB and ABA treatments. Anthocyanin content and expression levels of *VvMYBA1* and *VvUFGT* continued to increase with berry ripening (**Figures 3A,B**). Similarly, the expression of *VvVHP1;3* and especially *VvVHP1; 2* continuously increased with ripening; in contrast, the expression of *VvVHP1;1* and *VvVHP2* was not clearly altered during ripening (**Figure 3C**). When grape clusters were subjected to UVB treatment, it was found that UVB accelerated berry pigmentation and increased the expression of *VvMYBA1* and *VvUFGT* (**Figures 4A–C**; **Supplementary Figure S1A**). Consistently, *VvVHP1; 2* exhibited higher expression levels under UVB than under dark at all of the time points and additionally, its expression was gradually up-regulated by UVB from 84 h and reached the maximum at 156 h (**Figure 4E**). *VvVHP1;3* was induced, to lesser extents, by UVB from 44 h (**Figure 4F**). The two other *VvVHPs* were not induced by UVB (**Figure 4D, G**). When the berries were treated with exogenous ABA, berry pigmentation was accelerated, and the expression of *VvMYBA1* and *VvUFGT* was strongly induced compared to CK conditions (**Figures 4H–J** and **Supplementary Figure S1B**). Consistently, the expression of *VvVHP1; 2* was continuously induced by ABA and reached the peak at 10 days (**Figure 4L**). The other *VvVHPs* responded to ABA treatment in different manners, all of which were not consistent with the changing pattern of anthocyanins (**Figures 4K,M,N**). Therefore, the high associations of *VvVHP1; 2* expression with the expression of *VvUFGT* and *VvMYBA1* as well as with anthocyanin accumulation were found under normal, UVB and ABA conditions, suggesting the positive role of *VvVHP1; 2* in regulating anthocyanin accumulation.

### *VvVHP1; 2* Is Transcriptionally Activated Directly by VvMYBA1

**FIGURE 5:**
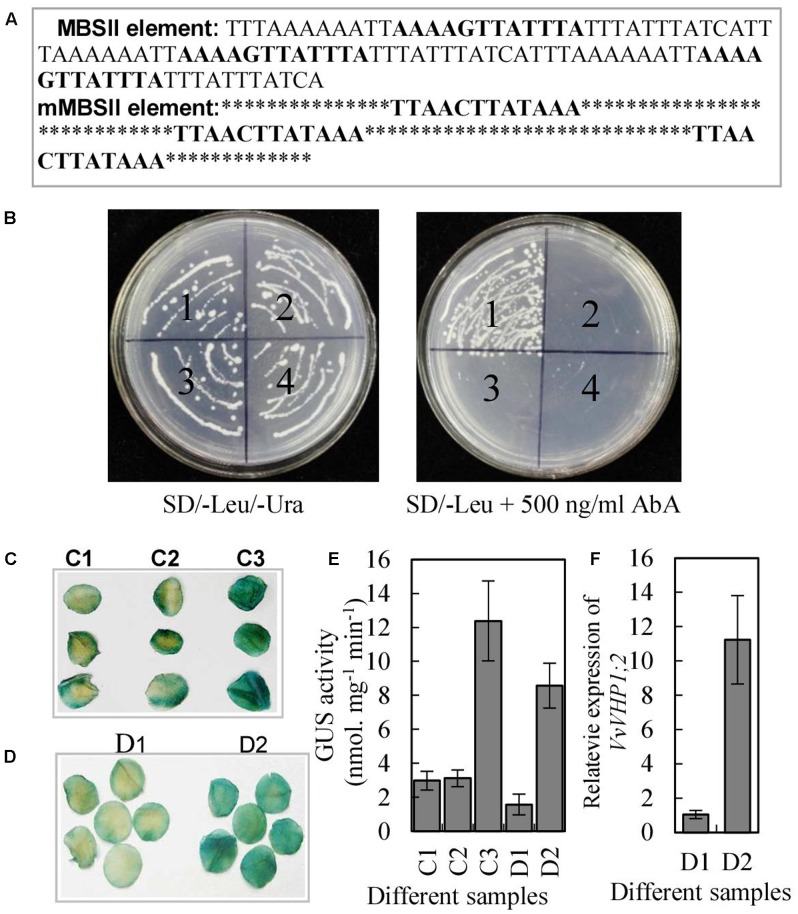
Characterization of transcription activation of *VvVHP1; 2* by VvMYBA1. **(A)** The sequence of the triple tandem repeats of MBSII and mMBSII binding elements. **(B)** Yeast one-hybrid assay using the 3 × MBSII and mMBSII as bait. Yeast cells carrying pGAD-MYBA1 or pGAD were grown on SD/-Leu/-Ura or SD/-Leu containing 500 ng.ml^-1^ AbA. (1) pAbAi-MBSII/pGAD-MYBA1; (2) pAbAi-MBSII/pGAD7; (3) pAbAi-mMBSII/pGAD-MYBA1; (4) pAbAi-mMBSII/pGAD7. **(C,D)** Histochemical analysis of the transactivation activity of VvMYBA1 via binding MBSII element. Round slices from 9-week-old tobacco leaves were *agro*-infiltrated with MBSII-35S mini-GUS alone (C1), with mMBSII-35S mini-GUS and 35S::MYBA1 (C2), with MBSII-35S mini-GUS and 35S::MYBA1 (C3), with P_V HP1;2_::VHP1;2-GUS (D1), and with P_V HP1;2_::VHP1;2-GUS and 35S:MYBA1. GUS staining was performed 2 days after the transformation. **(E)** GUS activities of the tobacco leaves infiltrated by *agrobacterium* containing different constructs. **(F)** *VvVHP1; 2* expression in the tobacco leaves infiltrated by *agrobacterium* containing D2 and D2 constructs (D1 and D2 were denoted in **D**).

The MBSII element in the promoter of *VvVHP1; 2* and the high correlation between *VvVHP1; 2* and *VvMYBA1* expression suggested that *VvVHP1; 2* transcription is likely to be activated by VvMYBA1. To verify this hypothesis, a yeast one-hybrid assay was performed to determine whether the VvMYBA1 protein was able to bind DNA. Three tandem repeats of MBSII binding sites or mutant MBSII binding sites were inserted into the pAbAi vector (**Figure 5A**), which harbors the AbA^r^ (aureobasidin^r^) reporter gene, and the corresponding constructs were designated pAbAi-MBSII and pAbAi-mMBSII. The two constructs were integrated into the genome of the yeast strain Y1HGold. AbA^r^ basal expression assays showed that 500 ng.ml^-1^ AbA could completely suppress the basal expression of pAbAi-MBSII reporter strain in the absence of prey. The full-length coding sequence of *VvMYBA1* was subsequently cloned into the yeast expression vector pGADT7, which harbors the GAL4 activation domain. The resulting pGADT7-MYBA1 and pGADT7 constructs were transformed into the yeast strain Y1HGold carrying the pAbAi-MBSII or pAbAi-mMBSII plasmids. All of the transformed yeast cells grew on leucine (Leu) and uracil (Ura), confirming the success of transformation (**Figure 5B**). As expected, only the yeast clones with pAbAi-MBSII and pGADT7-MYBA1 grew on SD/-Leu medium containing 500 ng. ml^-1^ AbA, suggesting that VvMYBA1 bound to the MBSII element and activated transcription in this yeast system.

To investigate whether VvMYBA1 activated gene expression by interacting with the MBSII element in plant cells, *Agrobacterium*-mediated transient expression of a *GUS* reporter gene in tobacco leaves was performed. The leaves co-transformed with MBSII-35S mini-GUS and 35S:MYBA1 had bluer color and higher GUS activity than those leaves transformed with mMBSII-35S mini-GUS and 35S:MYBA1 or only MBSII-35S mini-GUS (**Figures 5C,E**), indicating that the GUS reporter gene was activated in tobacco leaves due to the interaction between MYBA1 and the MBSII element. Additionally, the tobacco leaves co-transformed with P_V HP1;2_::VHP1;2-GUS (*VHP1;2-GUS* fusion gene driven by *VvVHP1; 2* promoter) and 35S:MYBA1 and those transformed with only P_V HP1;2_::VHP1;2-GUS were obtained. The results showed that the transcripts of *VvVHP1; 2* and GUS activity were positively regulated by VvMYBA1 (**Figures 5D–F**). Therefore, VvMYBA1 acts up-stream of *VHP1;2* to activate its transcript levels.

### Overexpression of *VvVHP1; 2* Positively Contributes to Anthocyanin Accumulation

**FIGURE 6:**
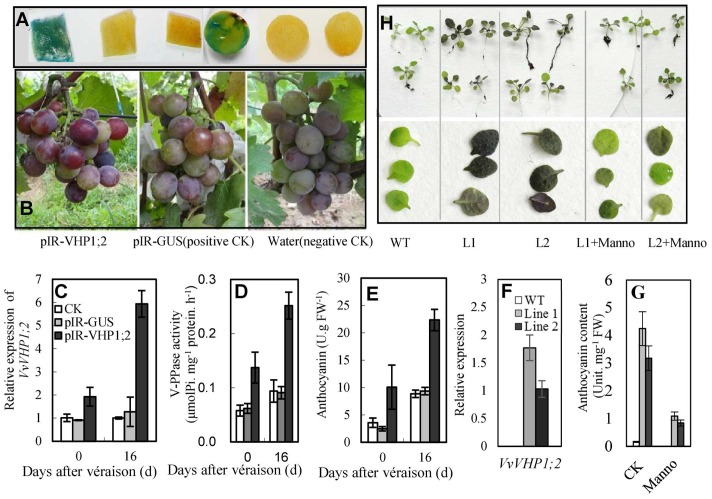
Overexpression of *VvVHP1; 2* promoted anthocyanin accumulations in transgenic berry skins **(A–E)** and *Arabidopsis* leaves **(F–H)**. **(A)** Histochemical GUS staining of pIR-GUS transfected berry skins and pulps. **(B–E)** Pigmentation at 0 DAV, gene expression, enzyme activity and anthocyanin content in berry skins at 0 and 16 DAV; the grape berries were transfected three times from –30 DAV at 3-day interval. **(F–H)** The expression, anthocyanin content and pigmentation in the 3-week-old WT and transgenic *Arabidopsis* leaves. The *Arabidopsis* seedlings were treated with 10-mM mannoheptulose or mannitol as an osmotic control from germination at 3-day intervals. Manno represents mannoheptulose.

To elucidate whether *VvVHP1; 2* could promote berry pigmentation, a viral transient expression system was employed to overexpress *VvVHP1; 2*. When IL-60-BS and pIR-GUS were co-administered to the grape berries, GUS activity was detected by staining in the berry skins and pulps (**Figure 6A**), indicating that the vector system worked in grape berries under the applied conditions. The transfection of pIR-VHP1;2 clearly promoted skin pigmentation and anthocyanin accumulation in the berry skins at the two sampling time points concomitantly with the enhanced *VvVHP1; 2* expression and total vacuolar PPase activities (**Figures 6B–E**). On the other hand, *VvVHP1; 2* was introduced into *Arabidopsis* to evaluate its *ex planta* functions. qRT-PCR with primers specific to *VvVHP1; 2* detected different levels of *VvVHP1; 2* transcripts in the leaves of two selected transgenic lines, while no *VvVHP1; 2* was detectable in the WT plants (**Figure 6F**), indicating that *VvVHP1; 2* was ectopically expressed in these transgenic *Arabidopsis* lines. Additionally, the leaves of the transgenic lines exhibited strong pigmentation and high content of anthocyanins compared to the WT controls (**Figures 6G,H**). Therefore, overexpression of *VvVHP1; 2* increased endogenous transcripts of *VvVHP1; 2* and VHP activities and thereby promoted anthocyanin accumulation.

### *VvVHP1; 2* Overexpression Promotes Anthocyanin Biosynthesis and Transport via Glucose Signal

**FIGURE 7:**
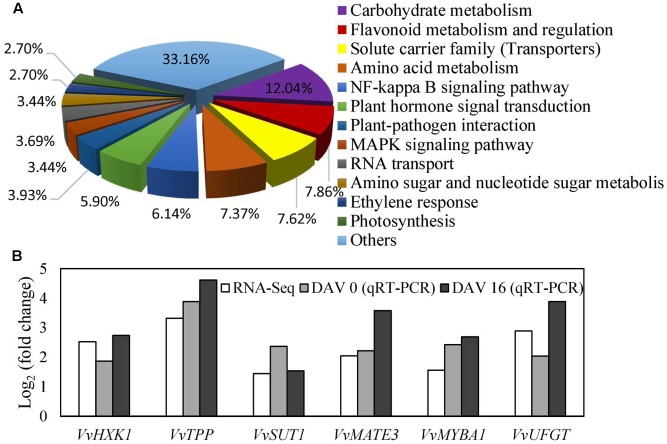
Biological processes of differentially expressed genes classified by gene ontology **(A)** and the most differentially expressed genes related to sugar signaling, anthocyanin biosynthesis and transport **(B)**. Fold changes from qRT-PCR at 0 and 16 DAV **(B)** were used to validate the DGE genes and to determine the expression changes of the DGE genes at different time points. Fold change from qRT-PCR was calculated by comparing the relative expression values of the selected genes in the transgenic and WT plants. Data are presented as the means of three replicates. Accession no of the genes are as follows: *VvHXK1*, XM_002283572; *VvTPP*, XP_002263078; *VvMATE3*, XM_002280387; *VvMYBA1*, AB097923; *VvUFGT*, AF000372.

To explore the mechanism underlying the regulation of *VvVHP1; 2* on pigmentation, RNA-Seq analysis of the WT and transgenic berry skins was performed to quantify gene changes. It was found that 354 and 409 genes were up- and down-regulated, respectively, in the transgenic skins (**Supplementary Table S2**). All of the annotated DGE genes were associated with 12 biological processes. The processes of carbohydrate metabolism, flavonoid metabolism and regulation, solute carrier family, amino acid metabolism and plant hormone signal transduction were clearly changed (**Figure 7A**). The processes of carbohydrate metabolism contained the most DGE genes, which are associated with glycolysis/gluconeogenesis, pentose and glucoronate interconversions, starch and sucrose metabolism, and so on (**Supplementary Table S2**), indicating the wide modification of carbohydrate metabolism by *VvVHP1; 2* overexpression. Particularly, the expression of *HXK1*, *trehalose-phosphate phosphatase* (*TPP*) and *sucrose-phosphate synthase*, which are involved in sugar signaling, were significantly up-regulated (**Supplementary Table S2**). A total of 32 genes involved in flavonoid metabolism and regulation were up-regulated, including *anthocyanidin 3-O-glucosyltransferase 5*, *anthocyanidin 5,3-O-glucosyltransferase*, *UFGT*, *MYBA1* and *MYBA2* (**Supplementary Table S2**). The solute carrier family contained 31 DGE genes, including *bidirectional sugar transporter SWEET15* and *SWEET 14*, *sucrose transporter 1* (*SUT1*), *glucose-6-phosphate translocator 2*, and *anthocyanin multidrug and toxic efflux transporter (MATE) 3* and *6* (**Supplementary Table S2**). Moreover, the expression levels of seven genes related to sugar signaling and anthocyanin biosynthesis and transport were detected by qRT-PCR, which validated that all of the detected genes were up-regulated to varying extents (**Figure 7B**), consistent with the RNA-Seq results. Therefore, pathways related to sugar signaling as well as anthocyanin biosynthesis and transport were altered by *VvVHP1; 2* overexpression.

**FIGURE 8:**
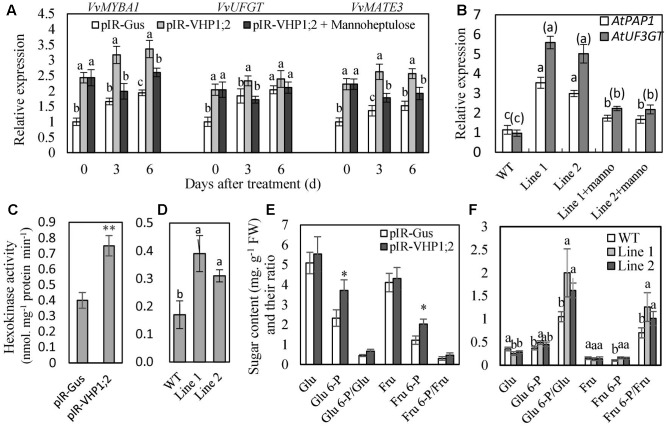
The expression levels of genes related to anthocyanin biosynthesis and transport **(A,B)**, hexokinase activity **(C,D)** and phosphorylated and non-phosphorylated sugar content **(E,F)** in the WT and transgenic berry skins and *Arabidopsis* leaves. Gene expression was detected in the *in vitro* cultured berry skins on MS medium containing 10 mM mannitol, as an osmotic control (pIR-GUS and pIR-VHP1;2), and mannoheptulose (pIR-VHP1;2+ Mannoheptulose) **(A)**, and in the leaves of *Arabidopsis* seedlings treated with mannoheptulose and mannitol **(B)**. Hexokinase activity **(C,D)** and sugar content **(E,F)** were determined in the berry skins at 0 DAV and 3-week-old *Arabidopsis* leaves. Values represent the means ± SD of 3 replicates. ^∗^significant difference; ^∗∗^highly significant difference. The difference was not significant at 5% significant level among the values labeled with the same letter. In **(B)**, letters and letters in brackets correspond to the comparison of *AtPAP1* and *AtUF3GT*, respectively; manno represents mannoheptulose. Accession no of the genes are as follows: *AtPAP1*, AF325123; *AtUF3GT*, NM_124785; the others are the same as **Figure 7**.

To further verify the regulation of glucose signaling by *VvVHP1; 2*, mannoheptulose, a specific inhibitor of HXK, was used to treat grape skins and *Arabidopsis* seedlings. The results showed that the mannoheptulose treatment reduced the *VvVHP1; 2* overexpression-induced increases in the expression of *VvMYBA1*, *VvUFGT* and *VvMATE3* in berry skins and *AtPAP1* and *AtUF3GT* in *Arabidopsis* leaves (**Figures 8A,B**). Additionally, anthocyanin content of transgenic *Arabidopsis* leaves was largely decreased by mannoheptulose treatment (**Figures 6G,H**). Therefore, the mannoheptulose treatment reduced the transcriptional up-regulations of the genes related to anthocyanin biosynthesis and transport and thereby decreased anthocyanin accumulation in the *VvVHP1; 2* overexpressing tissues, indicating that *VvVHP1; 2* regulated pigmentation via glucose signal.

In the meantime, total HXK activity as well as content of phosphorylated and non-phosphorylated glucose and fructose was determined (**Figure 8**). Overexpression of *VvVHP1; 2* led to significant increases of HXK activity in transgenic berry skins and *Arabidopsis* leaves when compared with WT controls (**Figures 8C, D**). The content of phosphorylated and non-phosphorylated glucose and fructose as well as their ratios were enhanced by *VvVHP1; 2* overexpression to varying extents and particularly, the increases of Glu 6-P and Fru 6-P reached significant levels in the transgenic berry skins (**Figure 8E**). In contrast, *VvVHP1; 2* overexpression reduced the content of glucose and fructose and significantly enhanced the content of Glu 6-P and Fru 6-P as well as the ratios of Glu 6-P/glucose and Fru 6-P/fructose in the transgenic *Arabidopsis* leaves (**Figure 8F**). Therefore, *VvVHP1; 2* overexpression increased HXK activity and modified the hexose status, which favored the generation of a sugar signal.

## Discussion

Two types of *VHPs* exist in plant cells. The number of *VHP1* and *VHP2* isoforms varies among different species. Three *VvVHP1s* and one *VvVHP2* were identified at the whole genome level in grape (**Figure 1A**); in contrast, two *VHP1s* and one *VHP2* were identified in cucumber (Kabała et al., 2014), one *AtVHP1* (also called *AVP1*) and two *AtVHP2*s in *Arabidopsis* (Segami et al., 2010), and 13 *VHPs* in cotton (Zhao et al., 2016). The difference in *VHP* number might result from the different copying rates of the whole genome and the duplication rates of *VHP* in evolutionary history (Zhao et al., 2016). Amino acid sequences between type I and II VHPs share low sequence similarities in *Arabidopsis* (Drozdowicz et al., 2000), cotton (Zhao et al., 2016), cucumber (Kabała et al., 2014) and grape (**Figure 1B**); less than 36% sequence identity was shared by AVP1 and AVP2 (Drozdowicz et al., 2000). Additionally, it is well known that *Arabidopsis* AVP1 exists primarily in the tonoplast, while AVP2 is localized to the Golgi apparatus and the trans-Golgi network and is absent in the tonoplast (Segami et al., 2010). The differences in amino acid sequence and subcellular location suggested different biological roles of the two types of VHPs. In contrast, amino acid sequences of different VHP1s are highly conserved in grape (**Figure 1A**), *Arabidopsis*, tobacco, rice and other species (Zhao et al., 2016). However, the different expression patterns of *VvVHP1;1*, *VvVHP1; 2*, and *VvVHP1;3* in roots, stems, leaves and fruits as well as under ABA and UVB treatments suggested their diverse functions. Similarly, wheat *TaVP1*, *2*, and *3* are differentially regulated spatially and in response to dehydration and salinity stresses; particularly, *TaVP3* is a seed development-specific gene in contrast to *TaVP1* and *2* (Wang et al., 2009); tomato *SlVP1* is constitutively expressed in almost all organs whereas the *SlVP2* transcript occurs at a higher level in young leaves (Mohammed et al., 2012); and cucumber *CsVHP1;1* was highly expressed in roots as well as in female flowers, while *CsVHP1;2* is not a tissue- or developmental stage-specific gene (Kabała et al., 2014). Additionally, the determinations of auxin-mediated organ development shows that the function of AVP1s are likely to be allele specific (Asaoka et al., 2016). Therefore, *VHPs* most likely participate in different biological processes rather than possess redundant functions.

*VvVHP1; 2* is suggested to be involved in mediating VvMYBA1-regulated berry pigmentation. The MYB/bHLH/WD40 complexes are thought to regulate the flavonoid pathway, and the MYB transcription factors determine the specificity of this complex and have been shown to directly bind to the structural gene promoters (Sainz et al., 1997). Microarray analyses of transgenic grapevines with altered expression of *VvMYBA1* show that *VvMYBA1* is a positive regulator of the later stages of anthocyanin biosynthesis including their glycosylation, methylation, acylation and transport into the vacuole (Rinaldo et al., 2015), and the expression of *vacuolar pyrophosphatase* (corresponding to *VvVHP1; 2* in our study) is significantly up-regulated in the *VvMYBA1* overexpressing ‘Chardonnay’ grape berries compared to non-transgenic controls (Rinaldo et al., 2015), suggesting the regulation by VvMYBA1 on *VvVHP1; 2*. Additionally, it was verified that *VvVHP1; 2* was a target gene of VvMYBA1 and could be directly activated by VvMYBA1 (**Figure 5**). On the other hand, it was reported that MdMYB1 binds to the promoters of two genes encoding the *B subunits* of *vacuolar H^+^-ATPase* to transcriptionally activate their expression, enhancing VHA activity, and thereby regulating anthocyanin and malate accumulation by directly facilitating their transport into vacuoles in apples (Hu et al., 2016). Similarly, the up-regulation of the solute transport carriers, including *MATE 3* and *6* (**Figure 7B** and **Supplementary Table S2**), suggested that *VvVHP1; 2* overexpression likely promote berry pigmentation via directly facilitating anthocyanin transport across the tonoplast. Therefore, *VvVHP1; 2* is transcriptionally activated by VvMYBA1 and hence promotes anthocyanin accumulation possibly by directly facilitating its transport into vacuoles.

*VvVHP1; 2* induced pigmentation via sugar signaling. It is remarkable that sugar-induced anthocyanin biosynthesis has been observed in many plant species. Glucose, fructose, and sucrose increased anthocyanin accumulation in an *in vitro* culture system of intact detached grape berries (Dai et al., 2014); sucrose is an important signal in the regulation of strawberry fruit ripening (Jia et al., 2013), and sucrose was the most effective inducer of anthocyanin biosynthesis in *Arabidopsis* seedlings (Solfanelli et al., 2006). HXKs are the first demonstrated intracellular glucose sensors in plants, and the functions of the HXK1 glucose sensor are likely evolutionarily conserved (Li and Sheen, 2016). In this study, the application of mannoheptulose, a specific inhibitor of HXK, indicated the role of *VvVHP1; 2* in regulating berry pigmentation via glucose signal. On the other hand, sucrose is sensed by the plant directly, through the generation of hexoses and through sugar signals such as trehalose-6-phosphate (Li and Sheen, 2016), and SUT as a potential sucrose sensor plays a key role in sucrose signaling (Barker et al., 2000). The significant up-regulation in the expression of *VvSUT1* and *VvTPP* (a key enzyme involved in trehalose-6-phosphate conversion) suggested that *VvVHP1; 2* is likely to affect sucrose signaling. Additionally, a few studies demonstrated that sugar-induced anthocyanin accumulation is suggested to result from altered expression of regulatory and structural genes, including *UFGT*, *DFR, LDOX* and *CHS*, and massive reprogramming in signaling transduction pathways (Solfanelli et al., 2006; Dai et al., 2014). The sucrose induction of anthocyanin biosynthetic genes may be attributed to the up-regulation of positive transcription factors such as *GL3*, *TT8* and *PAP1* concurrent with the downregulation of the negative transcription factor *MYBL2* (Jeong et al., 2010). Similarly, the enhanced anthocyanin in transgenic berry skin and *Arabidopsis* leaves (**Figures 6E,G**) might be attributed to the up-regulation of *VvMYBA1/AtPAP1*, *VvUFGT/AtUF3GT* and *VvMATE3* induced by*VvVHP1; 2* overexpression.

Sugar is a major component of fruit. Sucrose or glucose molecule is compartmentalized with its sensors/receptors in fruit tissue, cells or subcellular spaces; whether they play a regulatory role as a signal possibly depends on their change in content at action sites (i.e., at sites at which sensors/receptors exist), rather than their total content in whole fruit (Jia et al., 2013). The following pathways might be involved in *VvVHP1; 2*-induced changes of sugar status and generation of sugar signals. First, HXK1 plays dual roles in signaling and metabolism (Li and Sheen, 2016); the enhanced HXK activities (**Figures 8C,D**) led to the changes of phosphorylated and non-phosphorylated hexoses in the *VvVHP1; 2* overexpressing tissues (**Figures 8E,F**), which might further influence other sugar signal molecules, such as trehalose 6-P with Glu 6-P as a precursor (Griffiths et al., 2016). Therefore, *VvVHP1; 2* might affect sugar signal by HXK mediated changes of sugar status. Second, sugar signals originated from different sources, including active photosynthetic cells, dynamic storage reservoir, and organs for nutrient remobilization (Li and Sheen, 2016). It has been identified that *VHPs* overexpression can promote soluble sugar accumulation in berry skin (**Figure 8E**), sweet potato leaves (Fan et al., 2017) and apple callus (Yao et al., 2011). The enhanced sugar concentration in vacuoles might elicit feedback signaling in the photosynthetic source to ensure the balance of carbon flow (McCormic et al., 2009). Therefore, *VvVHP1; 2* might modify sugar signals by altering the dynamic storage of sugar. Third, SUT1 (also designated SUC1) plays a critical role in sucrose phloem loading and transport as well as cellular sugar partitioning (Fan et al., 2017); loss of *SUT1* function results in reduced sucrose transport, while increased *SUT1* expression results in increased sucrose transport (Kühn and Grof, 2010). The expression of *SUT1* was up-regulated in the *VvVHP1; 2*-overexpressing berry skins, *IbVP1*-overexpressing sweet potato (Fan et al., 2017) and *AVP1*-overexpressing *Arabidopsis* (Gaxiola et al., 2012). Therefore, *VvVHP1; 2* might promote translocation of sucrose from source to sink tissues, at least partially, via up-regulation of *VvSUT1*, and thereby induced modifications of sugar status. Fourth, in addition to acidifying vacuoles, the hydrolysis of cytosolic PPi is another major function of *AVP1*/*FUGU5* in plants; the *Arabidopsis thaliana fugu5* mutant, defective in *AVP1*, contained ∼2.5-fold higher PPi and ∼50% less sucrose than the wild type, indicating that PPi hydrolysis is essential for active gluconeogenesis to sustain post-germinative growth of *Arabidopsis* seedlings (Ferjani et al., 2011). Therefore, it is likely that *VvVHP1; 2* affects sugar signals by activating gluconeogenesis and thereby modifying sugar status.

## Conclusion

**FIGURE 9:**
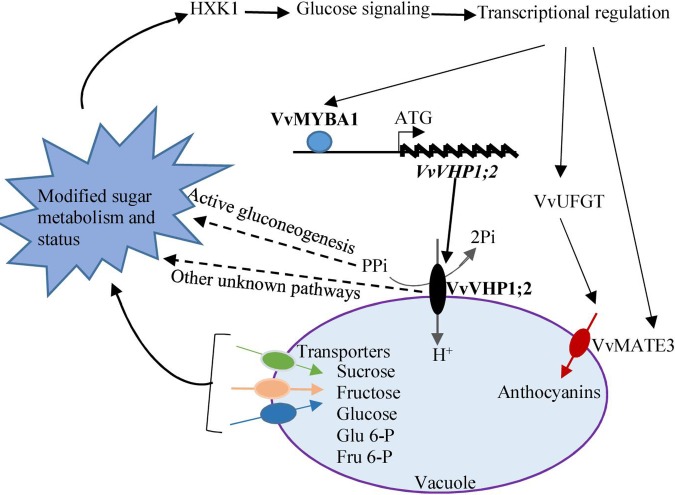
A postulated model of how *VvVHP1; 2* mediates the VvMYBA1-regulated berry pigmentation in grape berry skin. VvMYBA1 binds to the promoter of *VvVHP1; 2* and enhances its action. The enhanced activity of VvVHP1; 2 provides more energy for sugar transport across the tonoplast and thereby changes the balance of carbon flow. The enhanced activity of VvVHP1; 2 promotes the hydrolysis of cytosolic PPi, which is essential for active gluconeogenesis (Ferjani et al., 2011). Some other unknown pathways might participate in the *VvVHP1; 2* induced changes in sugar metabolism. Through these mechanisms, VvVHP1; 2 modifies sugar status and elicits glucose signal, which is sensed by HXK1. The genes related to anthocyanin biosynthesis and transport are transcriptionally up-regulated by glucose signal and thereby anthocyanin accumulation is promoted. VvMYBA1 directly transcriptionally activates the expression of *VvVHP1; 2* and in turn the glucose signal elicited by VvVHP1; 2 enhanced the *VvMYBA1* expression, which likely strengthens the process of the VvVHP1; 2-mediated berry skin pigmentation. Solid lines show experimentally demonstrated process, while dashed lines represent hypothetical process.

Taken together, the expression of *VvVHP1; 2* among the four identified *VvVHPs* correlated to berry skin pigmentation, and further experiments elucidated that *VvVHP1; 2* overexpression promoted anthocyanin accumulation in berry skins and *Arabidopsis* leaves. *VvVHP1; 2* was transcriptionally activated directly by VvMYBA1, and the increases in gene expression and enzyme activity of VvVHP1; 2 elicited glucose signal via modifying sugar status. The glucose signal from *VvVHP1; 2* overexpression up-regulated the expression of regulatory and structure genes related to anthocyanin biosynthesis and transport and thereby promoted anthocyanin accumulation in berry skin (**Figure 9**).

## Author Contributions

YY and HZ designed and supervised the research. TS, LX, HS, and QY carried out the experiments. TS and HS performed data analysis. YY wrote the paper. All authors reviewed the manuscript.

## Conflict of Interest Statement

The authors declare that the research was conducted in the absence of any commercial or financial relationships that could be construed as a potential conflict of interest.

## Footnotes

1 http://genomes.cribi.unipd.it/grape/

2 http://bioinformatics.psb.ugent.be/webtools/plantcare/html/

3 http://genomes.cribi.unipd.it/grape/

## Supplementary Material

**TABLE S1 |** Primers used in this study.

**TABLE S2 |** RNA-Seq profiles of the WT and transgenic berry skins.

**FIGURE S1 |** Berry pigmentation at different treatment time under UVB **(A)** and ABA **(B)** treatments.
