# Supplementary material for: VvVHP1; 2 Is Transcriptionally Activated by VvMYBA1 and Promotes Anthocyanin Accumulation of Grape Berry Skins via Glucose Signal
Source: Front Plant Sci. 2017 Oct 20;8:1811. doi: 10.3389/fpls.2017.01811 (PMC5655013; doi:10.3389/fpls.2017.01811)
Supplement: Supplementary file 1 [file Table_1.DOCX]

**Supplementary Table 1.** Primers used in this study.

| **Names of primer pair or genes** | **Forward primer (5' to 3')** | **Reverse primer (5' to 3')** | **Notes** |
| --- | --- | --- | --- |
| VHP-O1 | GCGTCGACATGGCGATTCTGTCAGATCT | GCGGATCCCTACTTCCAAATCTTGAAAAGCA | Amplifying the ORF of VvVHP1;2 |
| VHP-02 | GCGGATCCATGGCGATTCTGTCAGATCT | The same as VHP-01 | Amplifying the ORF of VvVHP1;2 |
| VHP-03 | GCGGATCCATGGCGATTCTGTCAGATCT | GCTCTAGACTACTTCCAAATCTTGAAAAGCA | Amplifying the ORF of VvVHP1;2 |
| VHP-P1 | F1: CCGTAACCATGGACAACC  F2: CGAAGCTTGCCTTTCAATTTTGAGGAATATTG | GCGGATCCCGGACCGACAAAATCCG | Amplifying the promoter of VvVHP1;2 |
| VHP-P2 | The same as VHP-P1 | GCTCTAGACGGACCGACAAAATCCG | Amplifying the promoter of VvVHP1;2 |
| MYB-ORF1 | GCGAATTCCGATGGAGAGCTTAGGAGTTAG | CCGAGCTCTCAGATCAAGTGATTTACTTGTGT | Amplifying the ORF of VvMYBA1 |
| *VvMYBA1* | GAGGGTGATTTTCCATTTGAT | CAAGAACAACTTTTGAACTTAAACAT | qRT-PCR |
| *VvUFGT* | GGGATGGTAATGGCTGTGG | ACATGGGTGGAGAGTGAGTT | qRT-PCR |
| *VvVHP1;1* | ACCTGAGCCGTTCTGTGG | CGTAGTAAAATCGCGGATCATC | qRT-PCR |
| *VvVHP1;2* | CCCGTCTCTCAACATCCTCATC | AAGCAGGCCACCATGTGC | qRT-PCR |
| *VvVHP1;3* | CTCACCAATTCATCTAATCTCC | TCTTCTGTGACAGTTAAAACCA | qRT-PCR |
| *VvVHP2* | GCTAAGTGTCCGGATTGATG | TGATCAACTTCCACAGAGTGA | qRT-PCR |
| *VvSUT1* | ATCTTGGCTATTCCTCGATCTA | TAACATTTAACCACCCATATTGA | qRT-PCR |
| *VvHXK1* | GGCCCATTGTATTTTGGTC | CCAACCGCATATCTGATCC | qRT-PCR |
| *VvTPP* | GCATTCCAACCGCATATCT | TCAGATCATTTGGAGCGTTAAC | qRT-PCR |
| *VvMATE* | GAAACCTTCTGAGTAAAGGCAG | CAATGCAATGATGAATGGAG | qRT-PCR |
| *AtPAP1* | GAGGTAGATATTTTGGTTCCTG | GTCTCTCCATCGAAAAGACTC | qRT-PCR |
| *AtUF3GT* | GTTAACGAACGGTTGTGGTTAG | GTGGAGATGTGTTTTGACTGAC | qRT-PCR |

[Restriction Enzyme cutting site](http://www.biodic.cn/detail.asp?id=452197)s are underlined.
