# Supplementary figures and images for: VvVHP1; 2 Is Transcriptionally Activated by VvMYBA1 and Promotes Anthocyanin Accumulation of Grape Berry Skins via Glucose Signal
Source: Front Plant Sci. 2017 Oct 20;8:1811. doi: 10.3389/fpls.2017.01811 (PMC5655013; doi:10.3389/fpls.2017.01811)

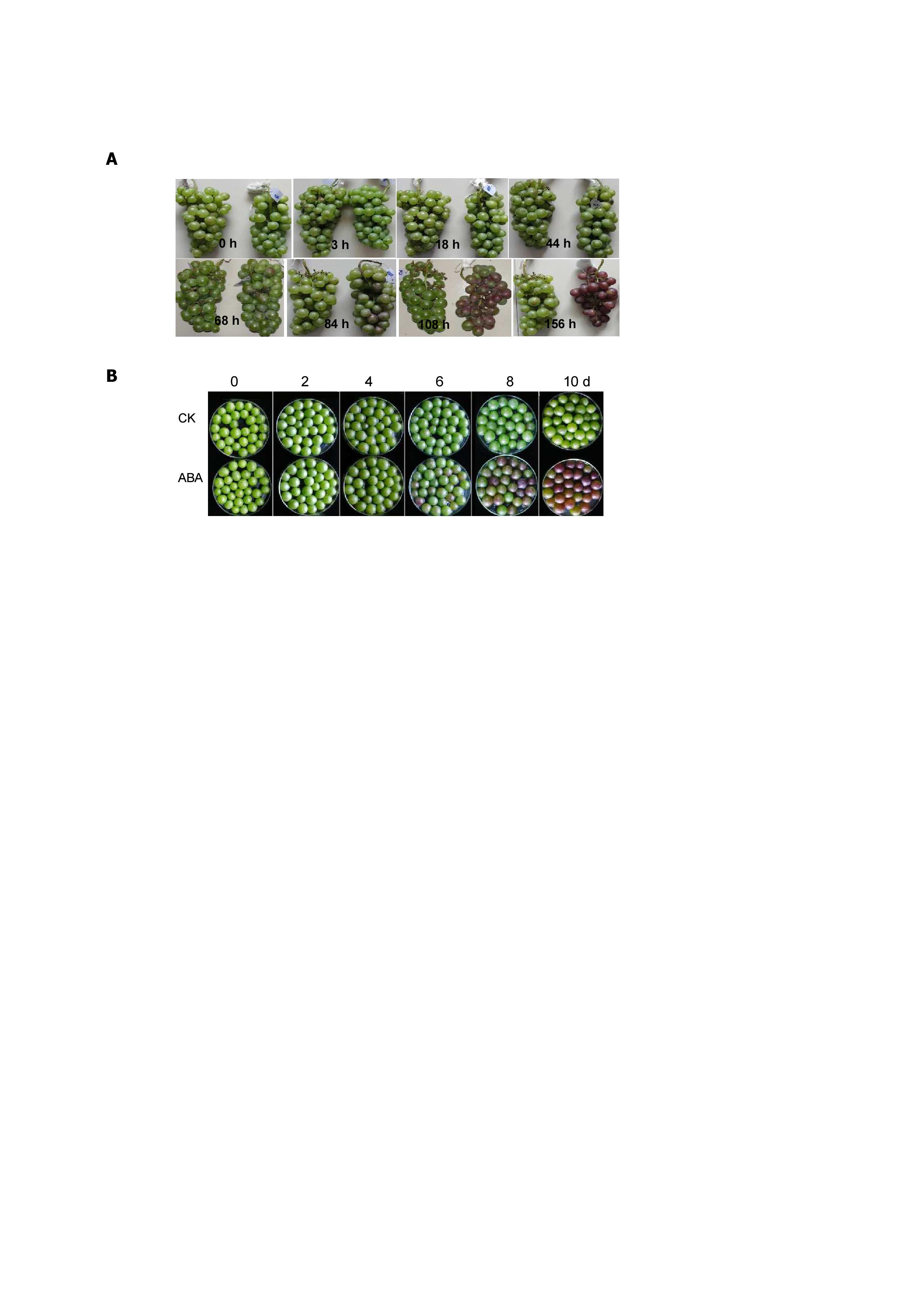

Supplement: Supplementary file 3 [file Image_1.JPEG]
